# Responsiveness of measures of heartburn improvement in non-erosive reflux disease

**DOI:** 10.1186/1477-7525-5-32

**Published:** 2007-06-11

**Authors:** Ola Junghard, Katarina Halling

**Affiliations:** 1 Biostatistics, AstraZeneca R&D, 431 83 Mölndal, Sweden; 2 Outcomes Research, AstraZeneca R&D, 431 83 Mölndal, Sweden

## Abstract

**Background:**

When measuring treatment effect on symptoms, the treatment success variable should be as responsive as possible. The aim of the study was to investigate the responsiveness of various treatment success variables in patients with symptoms of heartburn.

**Methods:**

A total of 1640 patients with non-erosive reflux disease (NERD) were treated with proton pump inhibitors for 4 weeks. Treatment success variables were based on a symptom questionnaire (Gastrointestinal Symptom Rating Scale) and on investigator-assessed heartburn, measured at baseline and after 4 weeks of treatment. The rates of treatment success were compared with patients' perceived change in symptoms, assessed by the Overall Treatment Effect questionnaire.

**Results:**

Generally, more stringent treatment success criteria (i.e., those demanding the better response) translated into more responsive treatment success variables. For example, the treatment success variable 'no heartburn' at 4 weeks was more responsive than the variable 'at most mild heartburn' at 4 weeks. Treatment success variables based on change from baseline to 4 weeks were, in general, less responsive than those based on the week 4 assessments only.

**Conclusion:**

In patients with NERD, responsiveness varied among different treatment success definitions, with more demanding definitions (based on the 4-week assessment) giving better responsiveness.

## Background

The resolution and enduring relief of symptoms in patients with gastro-esophageal reflux disease (GERD) is an important treatment goal [1]. Accurate symptom assessment is of particular importance in patients with non-erosive reflux disease (NERD), where symptom measurement is the sole method of evaluating the effectiveness of therapy. To be useful as endpoints in clinical trials, such measures should be responsive to change, i.e., they should reflect patients' changes in their symptom status and demonstrate responsiveness to treatment induced changes over time [2,3].

In clinical trials, symptoms are most often recorded on a scale, graded from 'no symptoms' to 'very severe symptoms', in 4 to 7 categories. These assessments are made either by the patient, in a diary card or in a questionnaire, or by an investigator. Such symptom scales may be treated as continuous variables by scoring the categories (e.g., 'no symptoms' = 0, 'mild symptoms' = 1, etc.) but they can also be used to define a dichotomised treatment success variable. When a scale is treated as a continuous variable, responsiveness may be evaluated using indicators such as the effect size and the standardised response mean, or by applying anchor-based methods [3]. Evaluation of responsiveness of symptom scales for upper gastrointestinal symptoms has been reported for scales treated as continuous variables [4-6].

From these symptom scales various treatment success variables may be derived, e.g., complete symptom resolution (i.e., no post-treatment symptoms) or at least one score point improvement since pre-treatment. When comparing treatments in a clinical trial, the results are presented as percentage of patients with treatment success, e.g., percent patients with complete symptom resolution, in each treatment group. Such results may be easier to interpret and communicate than results presented as a mean score change on a symptom scale.

This study examined responsiveness of different treatment success variables, with regard to heartburn, in patients with NERD.

## Methods

### Patients

Data were collected from patients who had experienced heartburn as their main GERD symptom for ≥ 6 months. Patients were enrolled if they had suffered heartburn for ≥ 4 days in the week prior to starting the study and had to have normal endoscopy results (i.e., no esophageal breaks) within 14 days of starting treatment.

### Study design and assessments

Patient data were pooled from two different studies of similar design and identical entry criteria [7]. In Study A, patients had received once-daily treatment with esomeprazole 40 mg, esomeprazole 20 mg or omeprazole 20 mg and in Study B they had received once-daily treatment with either esomeprazole 20 mg or omeprazole 20 mg. Overall heartburn severity (none, mild, moderate or severe) and the number of days with heartburn, both referring to the last 7 days, were assessed by the investigator at baseline and after 4 weeks of treatment.

Additionally, at baseline and after 4 weeks of treatment, patients answered the Gastrointestinal Symptom Rating Scale (GSRS) questionnaire, which includes 15 items [8]. The items are grouped into five domains, one of which is the Reflux domain, composed of a heartburn item and a regurgitation item. However, investigator assessment of both frequency and severity was done only for heartburn. In order to be comparable with this assessment the GSRS Heartburn item was chosen for this analysis of responsiveness. With reference to the previous 7 days, GSRS uses a Likert scale to assess symptom severity. Categories are scored from 0 to 6: 'no discomfort at all', 'minor', 'mild', 'moderate', 'moderately severe', 'severe', or 'very severe discomfort'.

After 4 weeks of treatment, patients also answered the Overall Treatment Effect (OTE) questionnaire, a questionnaire adapted from the Global Ratings of Change Questionnaire (GRCQ) with the permission of McMaster University, Hamilton, Ontario, Canada [9]. In this questionnaire, patients rated change in heartburn and regurgitation since start of treatment as being 'worse', 'unchanged' or 'better' since study start. If better, then the degree of improvement was rated in categories: 'almost the same, hardly better at all', 'a little better', 'somewhat better', 'moderately better', 'a good deal better', 'a great deal better' or 'a very great deal better'. If worse, the degree of deterioration was rated in a corresponding way. For the purpose of this analysis the original categories were collapsed into the following OTE groups: 'worse' (all 'worse' categories in OTE), 'unchanged', 'somewhat better' ('almost the same', 'a little' and 'somewhat' better categories in OTE), 'a good deal better' ('moderately' or 'a good deal' better categories in OTE), 'a great deal better' and 'a very great deal better'.

The OTE measures refers to change in both heartburn and regurgitation, but heartburn is the dominating symptom. Both at baseline and after 4 weeks, less than 8% of the patients had more severe regurgitation than heartburn. The change rated by OTE should thus mainly reflect changes in heartburn.

### Responsiveness

If a patient's health status changes over time, and a variable is able to reflect these changes, then this variable is responsive to change. In this evaluation of responsiveness we used the OTE subgroups described above for assessing the change in patients' health status. For continuous variables the magnitude of the effect size or standardised response mean may be used as a quantitative measure of responsiveness. Here we examined dichotomous treatment success variables and instead looked at the proportion of patients with treatment success in the different OTE subgroups.

For effective treatments where a relatively large number of patients get 'a very great deal better', a quantitative measure comparing responsiveness of treatment success variables may be the difference in treatment success rate between patients recording 'unchanged' and those recording 'a very great deal better' on the OTE questionnaire. The greater this difference, the better the responsiveness of the variable. There should also be a consistent increase in treatment success rate as the level of improvement increases. Thus, for patients who state that their symptoms have improved since pre-treatment, there should be a larger treatment success rate than for patients who state that their symptoms have not improved.

### Statistical analysis

The following treatment success variables, based on the 4-week assessment only, were examined:

- Based on the Heartburn item of GSRS: 3 success variables, defined as 'no heartburn', 'at most minor heartburn', and 'at most mild heartburn'

- Based on investigator-assessed heartburn severity: 2 success variables, defined as 'no heartburn' and 'at most mild heartburn'

- Based on investigator-assessed heartburn frequency: 3 success variables, defined as 'at most 1 day', 'at most 2 days' and 'at most 3 days' with heartburn during the last 7 days prior to the 4-week visit.

The following variables, based on improvement from baseline to 4 weeks, were also examined:

- Improvement in GSRS heartburn item by at least 1 score unit, at least 2 score units and at least 3 score units

- Improvement in GSRS Heartburn item by at least 50%

- Improvement in investigator-assessed heartburn severity by at least 1 grade

- Improvement in investigator-assessed heartburn frequency by at least 1, 2 and 3 days.

## Results

In total, data from 1640 patients with investigator assessment, GSRS and OTE recordings made on the same day were included in the analysis. Most patients had no heartburn at 4 weeks (61.3% according to investigator's assessment and 60.3% according to GSRS). Baseline demographics and clinical characteristics are summarised in Table 1.

**Table 1:** Baseline demographics and clinical characteristics of the pooled study population (n = 1640)

**Characteristic**	Patients (%)*
Male:Female	47:53
Age, years	
<50	54
50 to <65	34
≥ 65	12
History of heartburn episodes	
<12 months	11
1–5 years	35
>5 years	53
Overall heartburn severity during the previous 7 days (investigator-assessed)	
Mild	21
Moderate	62
Severe	18

*Values do not necessarily total 100% due to rounding.

**Figure 1:**
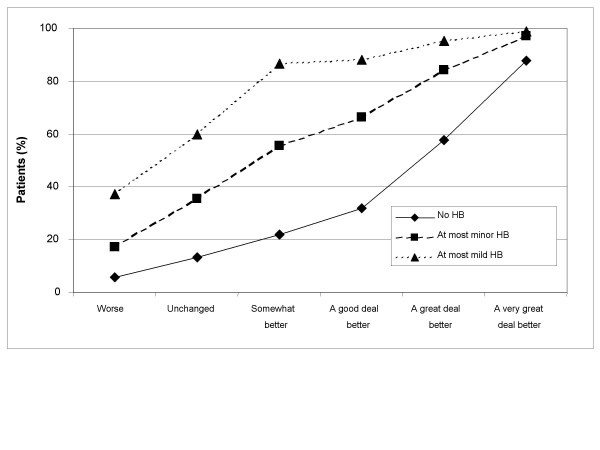
**Responsiveness of Gastrointestinal Symptom Rating Scale-assessed heartburn at 4 weeks**. Treatment success rate for variables based on the 4-week assessment of the GSRS Heartburn item, by Overall Treatment Effect classification after 4 weeks of treatment; HB, heartburn.

**Figure 2:**
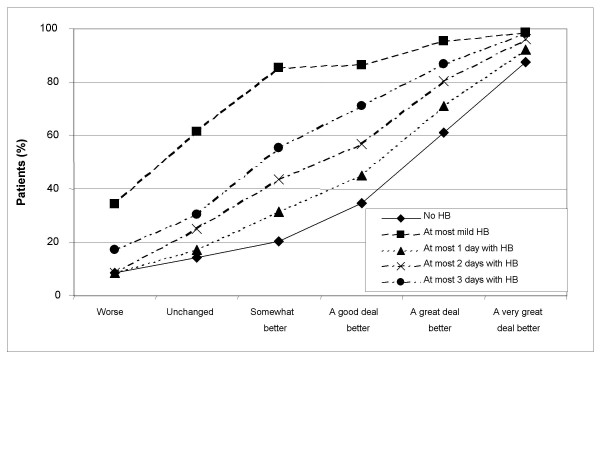
**Responsiveness of investigator-assessed heartburn at 4 weeks**. Treatment success rate for variables based on the 4-week investigator assessment of heartburn, by Overall Treatment Effect classification after 4 weeks of treatment; HB, heartburn.

**Figure 3:**
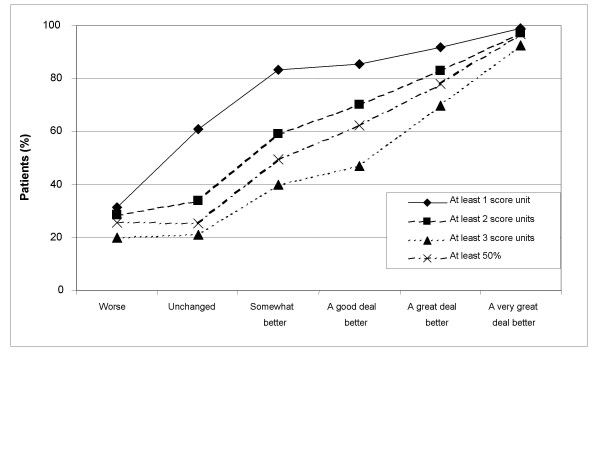
**Responsiveness of Gastrointestinal Symptom Rating Scale (GSRS)-assessed heartburn, in terms of improvement from baseline to 4 weeks**. Treatment success rate for variables based on improvement from baseline to 4 weeks according to the GSRS Heartburn item, by Overall Treatment Effect classification after 4 weeks of treatment.

**Figure 4:**
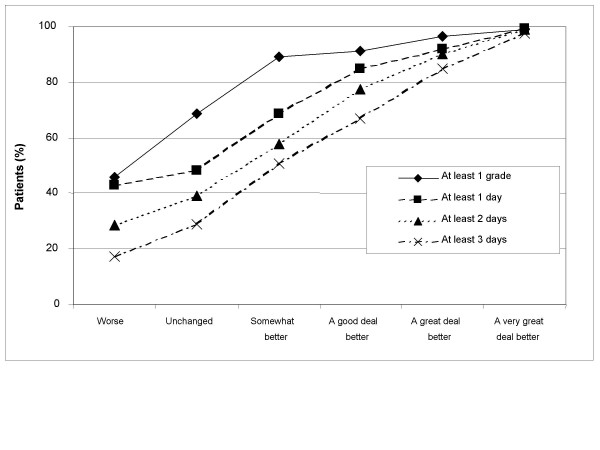
**Responsiveness of investigator-assessed heartburn, in terms of improvement from baseline to 4 weeks**. Treatment success rate for variables based on improvement from baseline to 4 weeks, according to investigators' assessment, by Overall Treatment Effect classification after 4 weeks of treatment.

Treatment success rates by OTE groups are presented numerically in Table 2 and graphically in Figures 1, 2, 3, 4. Treatment success variables based on the post-treatment assessments only are shown in Figures 1 (GSRS Heartburn item) and 2 (investigator-assessed heartburn). In general, the more stringent (or hard to achieve) the success criterion, the better the responsiveness as measured by the difference in success rates between patients being 'unchanged' and those being 'a very great deal better' according to OTE. For example, the difference in success rate between those who were 'a very great deal better' according to OTE and those who were 'unchanged' was 74.5 percentage points (87.8% minus 13.3%) for the variable 'no heartburn' according to GSRS Heartburn item (Figure 1 and Table 2). This difference decreased to 61.8 percentage points for the variable 'at most minor heartburn' and to 39.3 percentage points for the variable 'at most mild heartburn'. The general observation that a more stringent criterion gives better responsiveness in this population of patients also applied to success variables based on improvement from baseline, as can be seen in Figures 3 (GSRS Heartburn item) and 4 (investigator-assessed heartburn). For example, in Figure 3, the treatment success variable 'at least 1 score unit' improvement in GSRS Heartburn score is less stringent (or easier to achieve) than 'at least 2 score units' improvement, which in turn is less stringent than 'at least 3 score units' improvement. For these variables the difference in success rate between those who were 'a very great deal better' and those who were 'unchanged' was 38.2 percentage points, 63.4 percentage points and 71.4 percentage points, respectively.

**Table 2:** Percentage of patients with treatment success for heartburn by overall treatment effect group

	**Treatment success definition**						
	Worse (n = 35)	Unchanged (n = 181)	Somewhat better (n = 83)	A good deal better (n = 247)	A great deal better (n = 314)	A very great deal better (n = 780)	All (n = 1640)
**GSRS score at 4 weeks**							
0 (no HB)	5.7	13.3	21.7	32.0	57.6	87.8	60.3
≤ 1 (minor HB)	17.1	35.4	55.4	66.4	84.1	97.2	79.4
≤ 2 (mild HB)	37.1	59.7	86.7	88.3	95.2	99.0	90.4
**Inv-assessed severity**							
0 (no HB)	8.6	14.4	20.5	34.8	61.1	87.4	61.3
≤ 1 (mild HB)	34.3	61.3	85.5	86.6	95.5	98.7	90.1
**Inv-assessed frequency**							
≤ 1 day with HB	8.6	17.1	31.3	44.9	71.0	92.2	67.9
≤ 2 days with HB	8.6	24.9	43.4	56.7	80.3	96.2	74.8
≤ 3 days with HB	17.1	30.4	55.4	70.9	86.9	98.2	80.5
**No. of score units improved in GSRS**							
≥ 1	31.4	60.8	83.1	85.4	91.7	99.0	89.1
≥ 2	28.6	33.7	59.0	70.0	82.8	97.1	79.9
≥ 3	20.0	21.0	39.8	47.0	69.7	92.4	69.1
**≥ 50% improvement in GSRS**	25.7	25.4	49.4	62.3	78.0	96.8	76.2
**≥ 1 grade (Inv) improvement**	45.7	68.5	89.2	91.1	96.5	99.0	92.3
**Improvement in Inv-assessed frequency**							
≥ 1 day	42.9	48.1	68.7	85.0	92.0	99.4	87.4
≥ 2 days	28.6	39.2	57.8	77.3	90.1	98.8	83.8
≥ 3 days	17.1	28.7	50.6	66.8	85.0	97.7	78.9

GSRS = Gastrointestinal Symptom Rating Scale; HB = heartburn; Inv = investigator.

## Discussion

Responsiveness relates to the ability to detect changes when a patient improves or deteriorates. When choosing a treatment success variable to be used in a clinical trial, responsiveness is one of the key properties. Patients on a less effective drug will experience less improvement (which is captured by the OTE) than patients on a more effective drug. In order to make the difference in improvement as visible as possible in terms of a difference in treatment success rates, the treatment success variable should be as responsive as possible.

When treating patients with symptoms of heartburn with a proton pum inhibitor, the effect is rather dramatic. Among the patients examined in this study, 48% (780/1640, Table 2) rated their change as 'a very great deal better'. With almost half of the patients being 'a very great deal better' we believe that the difference in success rates between these patients and patients being 'unchanged' is a relevant measure of responsiveness when comparing different treatment success variables. In other situations, where patients experience a smaller change, other measures of responsiveness may be more relevant.

In evaluations of a clinically relevant change the categories 'almost the same, hardly better at all' and 'almost the same, hardly worse at all' are usually included in the 'unchanged' group. However, in this evaluation of responsiveness we did not include these categories in the 'unchanged' group. Few patients rated their change in these two categories (8 patients in total) and including these patients in the unchanged group would have had a minimal impact on the calculated success rates.

In this study, the treatment success variables with the best responsiveness were 'at most 1 day with heartburn' (investigator-assessed) and 'no heartburn' (investigator-assessed or according to GSRS) and these should be candidates for use in future GERD trials. The primary variable for the trials that were analysed in this study was in fact 'no heartburn' (according to the investigator assessment). Treatment success variables should also be realistic in order to be useful in everyday clinical life. For example, a patient reaching the criterion 'no heartburn' will have less symptoms than the healthy normal population where occasional heartburn is common. Allowing for one day per week with heartburn may be a more realistic outcome measure.

Change from baseline generally gave a lower responsiveness than 4-week assessments. If the heartburn response at 4 weeks depends on the baseline heartburn severity, in that patients with more severe heartburn tend to have mild heartburn after treatment, and patients with mild baseline heartburn tend to have no heartburn after treatment, a success variable based on change from baseline may be desirable. However, in these trials there was no such clear tendency. For example, the success rate of the 'no heartburn' variable was 61, 63 and 58 percentage points, respectively, for patients with mild, moderate and severe heartburn at baseline.

Previous studies on the responsiveness of symptom assessments for NERD have been made in terms of mean score of a symptom scale. This study has evaluated responsiveness in terms of percentage of patients with treatment success with regard to heartburn, and compared the responsiveness of different treatment success variables. One finding is that treatment success variables based on change from baseline to 4 weeks seem to be less responsive than those based on the week 4 assessments only; another finding is that more stringent treatment success criteria seems to translate into more responsive treatment success variables.

## Conclusion

To conclude, this study shows that responsiveness varied among different treatment success definitions with acid-suppressive therapy in the setting of NERD, with more stringent definitions based on the 4-week assessment giving better responsiveness.

## Competing interests

Both authors are full-time employees of AstraZeneca R&D, Mölndal, Sweden.

## Authors' contributions

Both authors were involved in study design and manuscript preparation. Data analysis was provided by OJ, and both authors were involved in data interpretation. Both authors read and approved the final submission.
